# Phytotoxicity of particulate matter from controlled burning of different plastic waste types

**DOI:** 10.1007/s00128-022-03581-9

**Published:** 2022-07-31

**Authors:** Katalin Hubai, Nora Kováts, Tsend-Ayush Sainnokhoi, Bettina Eck-Varanka, András Hoffer, Ádám Tóth, Gábor Teke

**Affiliations:** 1 Centre for Natural Sciences, University of Pannonia, Egyetem Str. 10, 8200 Veszprém, Hungary; 2 University of Pannonia, MTA-PE Air Chemistry Research Group, Egyetem Str. 10, 8200 Veszprém, Hungary; 3 ELGOSCAR-2000 Environmental Technology and Water Management Ltd, 8184 Balatonfűzfő, Hungary; 4 8200 Veszprém Egyetem Str. 10, Veszpr?m, Hungary

**Keywords:** Plastic waste, Illegal burning, Particulate matter, Phytotoxicity, Peroxidase

## Abstract

According to careful estimations, open burning of plastic waste affects app. 2 billion people worldwide. While human health risks have become more and more obvious, much less information is available on the phytotoxicity of these emissions. In our study phytotoxicity of particulate matter samples generated during controlled combustion of different plastic waste types such as polyvinyl chloride (PVC), polyurethane (PUR), polypropylene (PP), polystyrene (PS) and polyethylene (PE) was evaluated based on peroxidase levels. While different samples showed different concentration-effect relationship patterns, higher concentration(s) caused decreased peroxidase activities in each sample indicating serious damage.

## Introduction

The production of plastic waste poses a serious environmental health risk. Annually, over 400 million tons of plastic waste is generated (Hossain et al. 2021), of which less than 20% is reused (Liu et al. 2022). According to Velis and Cook (2021), worldwide app. 2 billion people burn their plastic waste in open fires. Open burning of plastic waste releases a variety of potentially harmful pollutants into the air such as persistent organic compounds, greenhouse gases and particulate matter (PM) (Cogut 2016). Uncontrolled burning is also a source of identified endocrine disrupting compounds (Sidhu et al. 2005). Toxic emissions results in serious human health problems including potential carcinogenic effects (e.g. skin cancer, lung cancer, leukaemia) and potential non-carcinogenic effects (e.g. liver and kidney damage, lung fibrosis, neurological damage, suppressed immune system, etc.) (Forbid et al. 2011). Human health effects are contributing to app. 200,000-270,000 premature deaths per year worldwide (Velis and Cook 2021).

While several studies have addressed the risk of these emissions to human health, much less information is available on their phytotoxicity. Plants are unwillingly exposed for shorter or longer periods, still potential damage posed by PM emission of waste burning has been very rarely addressed. As such, the main aim of the study was to evaluate phytotoxicity of PM emission from controlled burning of the following common plastic waste types: polyvinyl chloride (PVC), polyurethane (PUR), polypropylene (PP), polystyrene (PS) and polyethylene (PE). These particles bind potentially toxic compounds, of which heavy metals and polycyclic aromatic hydrocarbons (PAHs) are the most frequently addressed. PAHs originate from incomplete combustion processes which are quite typical considering burning conditions (Wu et al. 2021). Atmospheric PAHs are divided into gas and particle phases: those with less molecular weight are volatile and can be detected in the gas phase while those with high molecular weight will typically be absorbed by particulates (Ayyildiz and Esen 2020). These compounds pose the highest risk by producing reactive oxygen species (ROS) (Simões et al. 2021).

Phytotoxicity was assessed based on peroxidase (POD) content of test plants previously treated with the aqueous extract of PM_10_. As wet deposition is regarded an important exposure pathway (Grantz et al. 2003), this kind of treatment was meant to simulate wet deposition. POD is one of the earliest biomarkers reported for assessing impact of air pollution on plants (Keller 1974) and has proven generally reliable to indicate atmospheric particulate matter phytotoxicity (reviewed by Rai 2016). POD was found the most sensitive end-point in heavy-metal stressed experimental plants (Jaskulak et al. 2018) and gives a fast response to PAH exposure as well (Liu et al. 2009).

## Materials and methods

PM_10_ samples from the controlled burning of PVC, PUR, PP, PS and PE were collected on quartz filters. Detailed procedure and experimental conditions have been published in Hoffer et al. (2020). Aqueous extract was prepared as follows: each filter was cut into small pieces and placed in a beaker containing 200 mL high-purity water. The beaker was covered and kept at room temperature for 24 h. During that time, pieces were stirred several times. Finally the extract was filtered through 0.45-µm pore size filter and used immediately.

For POD measurement, white mustard (*Sinapis alba* L.) seedlings were grown according to the Phytotoxkit liquid samples seed germination and early growth of plants bench protocol (Microbiotests Inc. Belgium). Seed germination test is a widely accepted tool for evaluating waste incinaration ash leachate phytotoxicity (Ribé et al. 2014). In each concentration, 25 seeds were germinated at 25 °C for 3 days in Petri dishes covered by transparent cover in darkness. All concentrations were tested in 3 replicates. Peroxidase activity was measured as described previously (Kováts et al. 2021).

Analytical determinations were performed in the testing laboratory at the Laboratory of the ELGOSCAR2000 Environmental Technology and Water Management Ltd. accredited by the National Accreditation Authority under the registration number NAH-1-1278/2015. ICP-OES Thermo iCAP 6300 was used for heavy metal concentration determinations, according to EPA 6010 C: 2007. PAHs were measured by Agilent 6890GC 5973E MSD GC-MS, according to MSZ (Hungarian Standard) 1484-6:2003.

Analyses were carried out using oneway ANOVA, pairwise differences between treatment groups and control were calculated by Tukey HSD post-hoc tests in R Statistical Environment (R Development Core Team 2017).

## Results and discussion

**Fig. 1:**
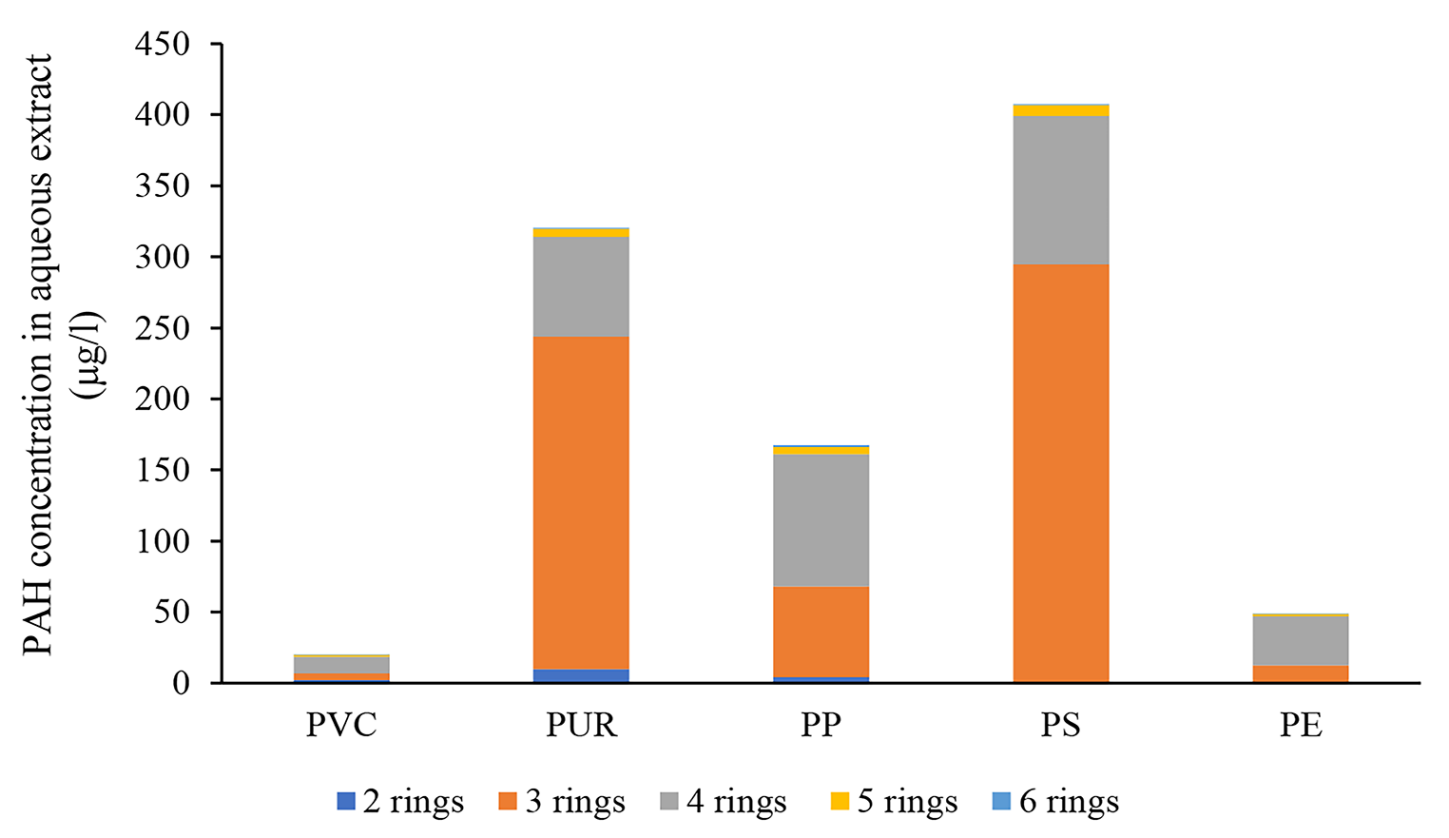
Amount of PAHs with different ring numbers in the aqueous extract

Concentration of different molecular weight PAHs groups in the samples is shown in Fig. 1. Potentially toxic heavy metal concentrations are discussed in case of each plastic sample.

**Fig. 2:**
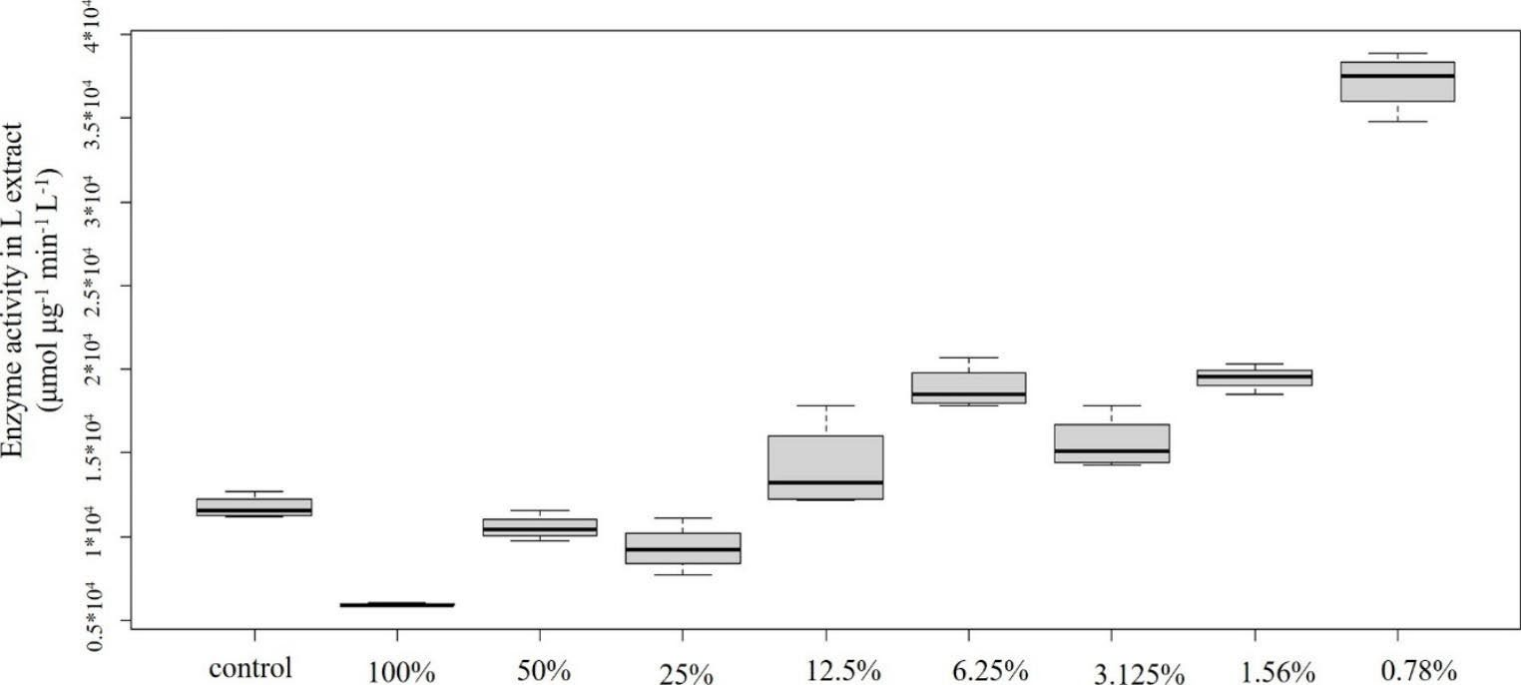
Concentration-effect relationship of PVC emission and POD level. Enzyme activity is given in µmol µg^− 1^ min^− 1^ L^− 1^ unit

**Fig. 3:**
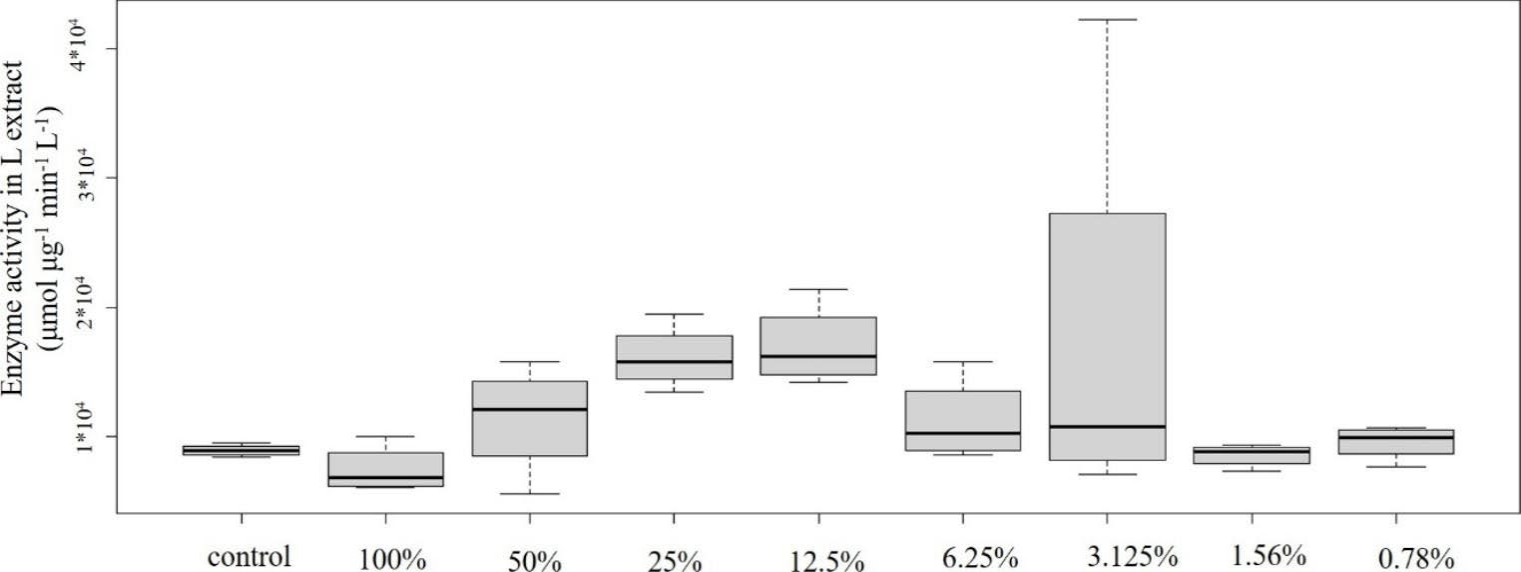
Concentration-effect relationship of PUR emission and POD level. Enzyme activity is given in µmol µg^− 1^ min^− 1^ L^− 1^ unit

**Fig. 4:**
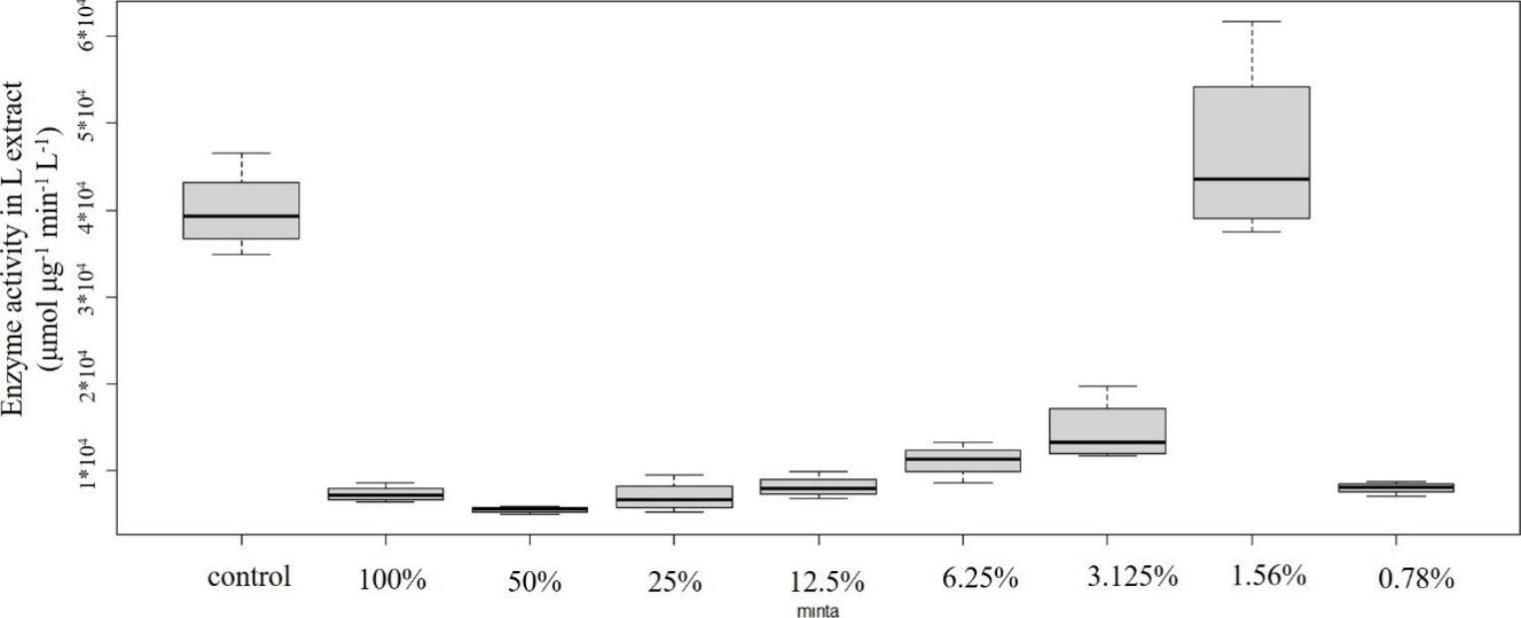
Concentration-effect relationship of PP emission and POD level. Enzyme activity is given in µmol µg^− 1^ min^− 1^ L^− 1^ unit

**Fig. 5:**
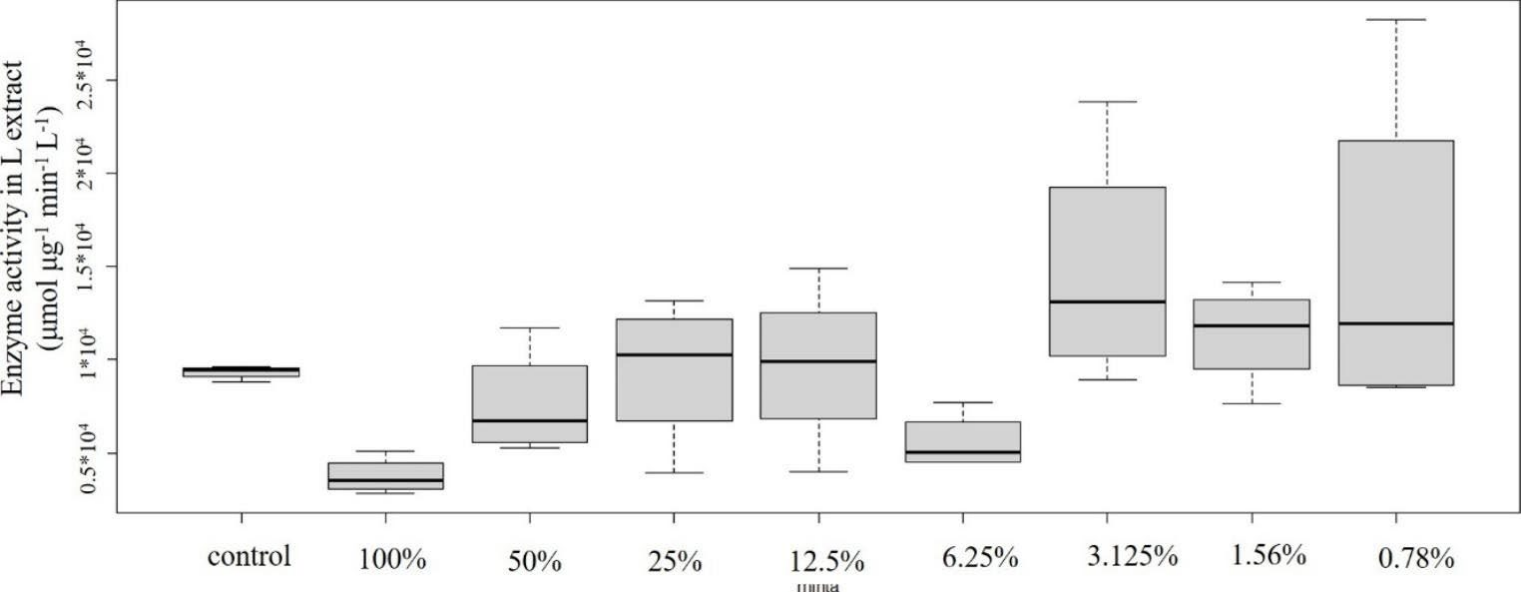
Concentration-effect relationship of PS emission and POD level. Enzyme activity is given in µmol µg^− 1^ min^− 1^ L^− 1^ unit

**Fig. 6:**
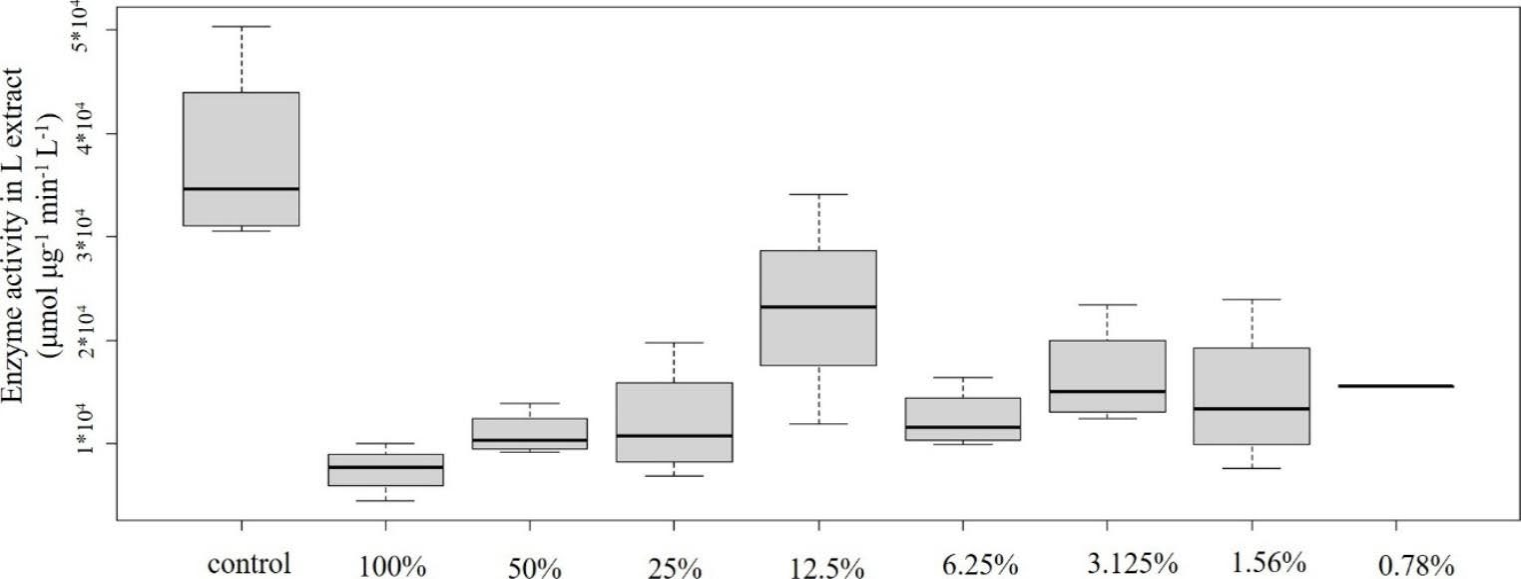
Concentration-effect relationship of PE emission and POD level. Enzyme activity is given in µmol µg^− 1^ min^− 1^ L^− 1^ unit

Concentration-effect relationships of plastic samples and POD enzyme activity are shown in Figs. 2, 3, 4, 5 and 6: Fig. 2 PVC; Fig. 3 PUR; Fig. 4 PP; Fig. 5 PS; Fig. 6 PE extracts.

### Polyvinyl chloride

The PVC sample contained high amount of Cd and Zn (22.4 µg/L and 78.8 µg/L). Other heavy metals present were Cu (6.26 µg/L), Ni (2.5 µg/L) and Mo (2.08 µg/L). It was the only sample which contained Pb above the detection limit (1.01 µg/L). Valavanidis et al. (2008) analysed chemical composition of PM from controlled combustion of different types of plastic and also detected high concentration of these metals in PVC samples. Cd is considered to be one of the most phytotoxic metals and is associated with a wide range of symptoms, and is highly responsible for ROS production (Akinyemi et al. 2017). Zn has also shown to induce oxidative stress and enhance the production of antioxidant enzymes (Passardi et al. 2005; Chemingui et al. 2019).

According to Tukey post hoc test significant differences were found between the control and the tested concentrations (Fig. 2). The 100% concentration showed statistically significant decrease in comparison to the control, than a gradual increase, finally the lowest concentration (0.78%) showed statistically significant increase. Peroxidase activity is reported by most of the studies to show concentration-dependent increase, not responding to low level of contamination (Mitrovic et al. 2004).

However, different patterns have also been reported. In the study of Huang et al. (2013) plants were treated with different concentrations of NH_4_^+^. Low levels did not cause significant changes in POD activity while it was significantly increased at higher concentrations. Finally, highest concentration of the treatment triggered significant decrease. The explanation may be the potential damage to cell and plasma membranes. It might be supposed that the 100% concentration in case of the PVC sample caused a serious damage to these vital membranes. Újvárosi et al. (2019) also observed decreased peroxidase activities in case of high level of stress and explained the phenomenon by the decreased capacity of scavenging reactive oxygen (ROS) species.

Several studies have discussed Cd-induced effects on biochemical markers. Similarly to our results, higher concentrations triggered the significant decrease in POD levels, indicating the damage to free radical metabolisms (Li et al. 2015). According to the authors, it might have resulted in the increase in relative cell membrane permeability. Reduced ascorbate peroxidase activity was measured in chromium treated basil (*Ocimum tenuiflorum*) plants in the study of Rai et al. (2004), indicating the sensitivity of the enzyme to this metal. Verma et al. (2008) also reported decrease in peroxidase activity in Cd-treated *Brassica* seedlings. On the other hand, lower concentrations result in the increased levels of POD and other antioxidant enzymes (e.g. Milone et al. 2003). Similar behaviour of peroxidases have been reported as a response to Zn (e.g. Yang et al. 2017, Ozdener and Aydin 2010). A strong correlation was found between Zn and POD activity (Hagemeyer 2004).

### 3.2 Polyurethane

Somewhat similar pattern could be detected in case of the PUR sample (Fig. 3). In comparison to the control, the 100% concentration caused significant decrease while the 25% and 12.5% concentrations elucidated significant increase in antioxidant capacity. Lower concentrations, however, did not trigger significant toxic effect comparing to the control. Similarly to the PVC sample, this sample also contained toxic heavy metals in detectable amount, though their concentration was lower than in the PVC sample (Cd 5.54 µg/L, Zn 11.9 µg/L). In addition to heavy metals, the sample contained high concentrations of PAHs as well (total PAH concentration was 321 µg/L) (Fig. 1).

### 3.3 Polypropylene

Concentration-effect relationship of the PP sample showed an ’all or nothing’ pattern (Fig. 4) (USEPA 2000): higher concentrations from 100 to 3.125% elucidated significant damage in stress enzyme activity while practically no response was detected in the next concentration (1.56%). Chemical analysis revealed the presence of toxic Cr and Zn (3.73 and 11.4 µg/L). Ni was detected in lower concentration, 2.02 µg/L. Zn-induced effects on POD has been discussed above. Cr was reported to elucidate the decrease in peroxidase activity as a result of inhibition of the major antioxidant metabolism (Choudhury and Panda 2005). According to Tiwari et al. (2013), Cr can have a negative effect on plant metabolism through inactivation of enzymes. In general, Cr-induced ROS can enhance membrane damage, degradation and deactivation of enzyme systems (Wakeel et al. 2020). The extract also contained relatively high concentration of PAHs (sum of PAHs was 167 µg/L). In addition to heavy metals and PAHs, Wu et al. (2021) detected several polychlorinated dibenzodioxin and dibenzofuran (PCDD/F) and polychlorinated biphenyl (PCB) congeners when PP containing sample was experimentally burned. The study also reported high cytotoxicity measured using human alveolar basal epithelial (A549) cell lines.

### 3.4 Polystyrene

Statistically significant difference (decrease of POD concentration) was found only in case of the highest concentration (Fig. 5), despite the fact that of all plastic samples investigated, particulates generated by this emission contained the highest amount of PAHs, 407 µg/L.

Effect of some of the PAHs being present in the extract have already been assessed on peroxidases or on other stress enzymes. Wei et al. (2014) e.g. reported the concentration-dependent increase in POD concentrations after phenanthrene (PHE) treatment. However, concentrations applied were significantly higher in comparison to our study: 0.05, 0.1, 0.2 mg/mL while the PS extract contained PHE in 213 µg/L concentration. Fluoranthene (FLT) had similar effect on POD activity in the study of Tomar and Jajoo (2015) but in a relatively high concentration, 5 mg/L. FLT concentration in the PS extract was 60.7 µg/L. Much less information is available on the combined effects of different PAHs. However, for example anthracene and5-ringbenzo[k]fluoranthene did not produce cumulative toxicity when applied together (Wieczorek et al. 2015; Radič et al. 2018) assessed the phytotoxicity of coal combustion polluted soil samples on the test plant *Lemna minor* but measured effects could not be statistically correlated to individual PAH compounds.

### 3.5 Polyethylene

Every concentration tested showed significant decrease in comparison to the control (Fig. 6), implying that membrane damage could be anticipated even at the lowest concentration. Analytical measurements covered only heavy metal and PAH compounds. Potentially toxic heavy metals being present in the extract were: Cu 6.72 µg/L, Zn 8.23 µg/L. Sum of PAHs was rather low (48.7 µg/L extract). Most possibly other compounds could also be responsible for the effect. Gullett et al. (2001) detected polychlorinated dibenzodioxin and dibenzofuran when PE containing domestic waste was experimentally burned. Mei et al. (2017) found significant emission of polybrominated dibenzo-p-dioxins/dibenzofurans during lab-scale pyrolysis of 90% PE and 10% decabromodiphenyl ether (deca-BDE). Polybrominated diphenyl ethers (PBDEs) are considered one of the new types of persistent organic pollutants (POPs). According to Sun et al. (2019), treatments with deca-BDE resulted in the decrease of POD activity in the freshwater test organism *Lemna minor*.

Conesa et al. (2009) reported considerable emission of volatile organic compounds (VOC) and semi-volatile compounds during the controlled combustion of PE, volatiles including benzene and toluene while semi volatiles including biphenyl in outstanding concentrations. These compounds have proven toxicity (e.g. Davidson et al. 2021; Williams et al. 2020). Benzene was classified as carcinogenic to humans (belonging to IARC group 1) already in 1979, on the basis of sufficient evidence that it causes leukaemia, reaffirming the classification specifically for acute myeloid leukaemia (AML) and acute non-lymphocytic leukaemia in 2009 (reviewed by Loomis et al. 2017).

## 4. Conclusions

As concluding remark, it should be noted that while concentration-effect graphs showed somewhat different pattern, highest concentration(s) triggered the damage of POD in each sample. Chemical analysis of the samples revealed that the samples can be characterised by high heavy metal or high PAH content, some samples are of mixed nature, containing both types of potentially toxic compounds. However, these groups of potentially toxic compounds could not explain resulting toxicity in all cases, especially for the polyethylene sample. In general, our results show the high phytotoxic risk generated by illegal burning of plastic waste, especially considering the fact that in ’everyday practice’ these plastic types are mixed, providing a complex toxic cocktail for exposed plants.
